# Evolution and Characterization of Acetyl Coenzyme A: Diacylglycerol Acyltransferase Genes in Cotton Identify the Roles of *GhDGAT3D* in Oil Biosynthesis and Fatty Acid Composition

**DOI:** 10.3390/genes12071045

**Published:** 2021-07-07

**Authors:** Yan-Peng Zhao, Na Wu, Wen-Jie Li, Jian-Ling Shen, Chen Chen, Fu-Guang Li, Yu-Xia Hou

**Affiliations:** 1 State Key Laboratory of Cotton Biology, Zhengzhou Research Base, School of Agricultural Sciences, Zhengzhou University, Zhengzhou 450001, China; ypzhao@zzu.edu.cn (Y.-P.Z.); w15093389925@163.com (N.W.); 13781949770@163.com (W.-J.L.); shenjianling8@163.com (J.-L.S.); C18337103690@163.com (C.C.); 2 State Key Laboratory of Cotton Biology, Institute of Cotton Research, Chinese Academy of Agricultural Sciences, Anyang 455000, China; 3 College of Science, China Agricultural University, No. 2 Yuanmingyuan West Road, Beijing 100193, China

**Keywords:** acyl-coenzyme A: diacylglycerol acyltransferase, *Gossypium*, GhDGAT3, upland cotton, oil biosynthesis

## Abstract

Cottonseed oil is rich in unsaturated fatty acids (UFAs) and serves as an edible oil in human nutrition. Reports suggest that acyl-coenzyme A: diacylglycerol acyltransferases (*DGAT*) and wax ester synthase/DGAT (*WSD1*) genes encode a key group of enzymes that catalyze the final step for triacylglycerol biosynthesis and enable an important rate-limiting process. However, their roles in oil biosynthesis and the fatty acid profile of cotton seed are poorly understood. Therefore, the aim of this study was to identify and characterize *DGAT* and *WSD1* genes in cotton plants and examine their roles in oil biosynthesis, the fatty acid profile of cotton seeds, and abiotic stress responses. In this study, 36 *GhDGAT* and *GhWSD1* genes were identified in upland cotton (*G*. *hirsutum*) and found to be clustered into four groups: GhDGAT1, GhDGAT2, GhDGAT3, and GhWSD1. Gene structure and domain analyses showed that the *GhDGAT* and *GhWSD1* genes in each group are highly conserved. Gene synteny analysis indicated that segmental and tandem duplication events occurred frequently during cotton evolution. Expression analysis revealed that *GhDGAT* and *GhWSD1* genes function widely in cotton development and stress responses; moreover, several environmental stress and hormone response-related *cis*-elements were detected in the GhDGAT and GhWSD1 promoter regions. The predicted target transcription factors and miRNAs imply an extensive role of *GhDGAT* and *GhWSD1* genes in stress responses. Increases in GhDGAT3 gene expression with increases in cottonseed oil accumulation were observed. Transformation study results showed that there was an increase in C18:1 content and a decrease in C18:2 and C18:3 contents in seeds of Arabidopsis transgenic plants overexpressing *GhDGAT3D* compared with that of control plants. Overall, these findings contributed to the understanding of the functions of *GhDGAT* and *GhWSD1* genes in upland cotton, providing basic information for further research.

## 1. Introduction 

Cotton (*Gossypium*) is the fifth largest oil crop in the world and the most important natural fiber producing plant. Among cotton species, the upland cotton (*Gossypium hirsutum*, AD_1_) is the most cultivated, accounting for over 90% of cultivated cotton species due to its high fiber production and widespread environmental adaptation. *G*. *hirsutum* is believed to have resulted from the hybridization and genome doubling of two diploid ancestors, *Gossypium arboreum* (A_2_) and *Gossypium raimondii* (D_5_) [1]. Cottonseed oil is the primary byproduct of cotton processing, accounting for approximately 16% of the seed’s weight [2]. Moreover, cottonseed oil is an excellent edible oil for human consumption due to its high content of unsaturated fatty acids (UFA), consisting of 57.64% linoleic acid (C18:2), 22.92% palmitic acid (C16:0), 15.39% oleic acid (C18:1), and 2.22% stearic acid (C18:0) [3]. Additionally, high unsaturated fatty acid content has been linked to a reduced risk of cardiovascular diseases. Additionally, the large amount of cottonseed oil serves as a valuable resource for bioenergy [4].

Oil biosynthesis in plants includes two steps: de novo fatty acid biosynthesis in plastids and triacylglycerol (TAG) biosynthesis in the endoplasmic reticulum (ER) [5,6]. Lipid droplets (oil bodies) are the main type of seed oil stored in plants, with TAGs surrounded by steroleosins, oleosins, and caleosins. During TAG biosynthesis, glycerol-3-phosphate (G3P) serves as the substrate, while acyl-CoA serves as the acyl donor. In this pathway, glycerol-3-phosphate acyltransferase (GPAT) catalyzes the first acylation of G3P to yield lysophosphatidic acid (LPA), which is further acylated by lysophosphatidic acid acyltransferase (LPAAT) to produce phosphatidic acid (PA), followed by the conversion of PA to diacylglycerol (DAG) by phosphatidic acid phosphatase (PP). DGAT catalyzes the final acylation of the sn-3 position of DAG to form TAG, which is the committed step in acyl-CoA dependent TAG biosynthesis, known as the Kennedy pathway [7,8]. However, an acyl-CoA independent pathway also exists, in which phospholipid: diacylglycerol acyltransferase (PDAT) transfers an acyl moiety from phosphatidylcholine to a DAG sn-3 position to produce TAG [9,10]. Overall, both DGAT and PDAT play crucial roles in guiding carbon flux into TAG.

DGAT has been elaborately characterized to play a crucial role in DAG-to-TAG transformation. Three types of *DGAT* genes have been identified in plants: ER-localized *DGAT1* and *DGAT2*, and soluble cytosolic *DGAT3* [11,12]. *DGAT1* is highly expressed in developing embryos of oilseed crops, and its expression level is correlated with oil accumulation during seed development [13,14]. Inactivation of *DGAT1* results in decreased seed oil content in the Arabidopsis mutant *AS11* [15]. *ZmDGAT1* activation (by inserting a phenylalanine) increases the oil content of a maize (*Zea mays*) embryo [16]. However, compared with the role of *DGAT1* in seed oil deposition, *DGAT2* plays a minor role in regulating oil production [17]. There are no differences between *AtDGAT2* mutants and the wild type (WT) in TAG accumulation [18]. However, *AtDGAT2* in TAG biosynthesis was functionally confirmed via transient expression in *Nicotiana benthamiana* leaves [19]. Overexpression of *Jatropha curcas JcDGAT1* or *JcDGAT2* resulted in a 25% and 29.6% increase in oil production, respectively, compared with that of control plants [20]. The plant soluble cytosolic *DGAT3* was first identified in peanuts (*Arachis hypogaea*) [21]. A study showed that Arabidopsis *DGAT3* participated in the collecting of C18:2 and C18:3 into TAG when TAG breakdown was blocked [22]. *AtDGAT3* is a metalloprotein involved in TAG biosynthesis [23]. Seed-specific expression of *Camelina sativa CaDGAT3-3* significantly enhanced oil and unsaturated fatty acid accumulation, with higher levels of eicosanoic acid in tobacco seeds [24]. The reports of a previous study showed that the expression of *GhDGAT3* was higher than that of *GhDGAT1* and *GhDGAT2* in developing cottonseeds, indicating the key role of *GhDGAT3* in oil biosynthesis [3]. 

Furthermore, another enzyme of interest in the cotton plant is the WSD1 enzyme. WSD1 has a high level of wax synthase activity and an approximately 10-fold lower level of diacylglycerol acyltransferase activity than that of wax synthase activity [25], which is necessary for protection against desiccation [26]. Moreover, the WSD1 enzyme has been reported to play a considerable role in environmental adaptation and stress management [26,27]. 

Genome-wide analysis of the DGAT gene family has been performed in maize [28], oil palms [29], and soybeans [14,30]. A previous study also compared the structure and motifs of DGATs in upland cotton with that of oil palms [29]; however, a comprehensive analysis is still lacking. Therefore, the aim of this study was to comprehensively examine and characterize the DGAT and WSD1 gene families and to identify their functions in upland cotton. Based on the updated genome data [31], we performed a characteristic analysis of GhDGAT and GhWSD1 family genes in upland cotton. *GhDGAT3D* was functionally confirmed by overexpression in Arabidopsis. Our study aimed to improve the understanding of the roles of *GhDGAT* in oil biosynthesis and the function of *DGAT* genes in other oil crops.

## 2. Materials and Methods 

### 2.1. Identification of DGAT and WSD1 Family Members in Gossypium

Genome datasets of *Gossypium hirsutum* acc. TM-1 (AD_1_, CRI_v1) and its ancestors, *G. arboreum* (A_2_, CRI_v1.0) and *G. raimondii* (D_5_, JGI_v2.1), were downloaded from the CottonGen website [32]. To identify candidate *DGAT* and *WSD* genes in cotton, the genome datasets were aligned against Arabidopsis DGAT and WSD amino acid (aa) sequences of *AtDGAT1* (*AT2G19450*), *AtDGAT2* (*AT3G51520*), *AtDGAT3* (*AT1G48300*), and *AtWSD1* (*AT5G37300*) for BLASTp search. The *DGAT* and *WSD1* genes were annotated according to their corresponding orthologs in Arabidopsis and chromosomal location in upland cotton, and genes in *G. hirsutum* were named based on their homoeologs in each subgenome, with ‘A’ and ‘D’ representing homoeologs in the At and Dt subgenomes, respectively [33]. Whether a gene was from an At subgenome or Dt subgenome was judged by their DNA sequence homology with an A genome diploid species (*G. arboreum*) or a D genome diploid species (*G. raimondii*). The theoretical molecular weight (MW) and isoelectric point (pI) were predicted using ExPASy software [34]. Subcellular localization of DGAT and WSD1 proteins was evaluated via the WoLF PSORT server (https://wolfpsort.hgc.jp/, accessed on 28 June 2021). The regions 2 kb upstream of the start codon of *GhDGAT* and *GhWSD1* promoter were subjected to the PlantCARE database [35] for cis-element searching.

### 2.2. Phylogenetic, Gene Structure, Conserved Domain, and Motifs Analysis

The muscle program in MEGA-X software [36] was used to perform multiple amino acid sequence alignments, and then the unrooted phylogenetic tree was constructed using the maximum likelihood (ML) method by bootstrap tests with 1000 replicates. The phylogenetic tree was visualized using the iTOL tool [37]. The structure of *GhDGAT* and *GhWSD1* genes was obtained from the genomic dataset and exhibited by using a gene structure display server (GSDS) [38]. The conserved domains of GhDGAT and GhWSD1 proteins were searched using SMART software [39] and displayed using TBtools [40]. Amino acid sequences of GhDGAT and GhWSD1 in the *G. hirsutum* protein dataset were submitted to the MEME program to identify the conserved protein motifs [41]. A functional search of the conserved motifs was performed using the InterProScan database. To determine the evolutionary relationships, the transmembrane (TM) structures of GhDGAT and GhWSD1 proteins were predicted using the TMHMM-2.0 website (http://www.cbs.dtu.dk/services/TMHMM/#opennewwindow, accessed on 16 June 2020).

### 2.3. Chromosomal Location and Gene Synteny Analysis

The physical location on the chromosome of *GhDGAT* and *GhWSD1* in *G. hirsutum* was acquired from the genomic datasets and displayed with Mapchart 2.2 software [42]. Gene synteny analysis was carried out using MCScanX software [43], and Circos [44] was used for graphical depiction.

### 2.4. Expression Pattern Analysis

The transcript level was calculated based on publicly released data. Gene expression datasets for developing cottonseeds were acquired by Hu et al [45]. RNA-Seq datasets of different tissues and cottonseed development in *G. hirsutum* were obtained from the BioProject with accession number PRJNA490626 [46]. Transcript levels were estimated as fragments per kilobase million (FPKM) value using HISAT and StringTie software tools [47].

### 2.5. Transcription Factors and miRNAs Targeting GhDGAT and GhWSD1 Homologs

Transcription factors regulating *GhDGAT* and *GhWSD1* were predicted using PlantRegMap [48], with *G. raimondii* as the target. The full-length cDNA sequences of GhDGAT and GhWSD1 homologs were submitted to the psRNATarget website [49] for a potential miRNAs search against the *G. hirsutum* miRNA database. The relationships between the predicted TFs and miRNAs with *GhDGAT* and *GhWSD1* were displayed by Cytoscape software [50].

### 2.6. Cotton Seedlings Treatments and Sampling

Seedlings of upland cotton, Zhongmiansuo 24 (ZM24) variety, were used for gene expression analysis in response to drought, salt, and cold stresses. Seedlings in three-leaf-stage were cultivated in Hoagland liquid medium containing 17% PEG6000 or 200 mM NaCl for drought and salt stresses, respectively, and were exposed to 4 °C conditions for cold stress. The leaves of the cotton seedlings were collected at eight time points (0, 0.5, 1, 3, 6, 12, 24, and 48 h) and rapidly frozen in liquid nitrogen for total RNA extraction.

### 2.7. Recombination Vector Construction and Arabidopsis Transformation

Based on the expression module, *GhDAGT3D* was selected for functional analysis via genetic transformation. The full-length cDNA of *GhDGAT3D* was cloned from the embryo of ZM24 upland cotton variety at 20 DPA. The ClonExpress^®^ MultiS One Step Cloning Kit (Vazyme, Nanjing, China) was used to clone GhDGAT3D into the pCAMBIA2300 vector behind a 35S promoter, which we named p35S::GhDGAT3D recombined vector in this study. The recombined vector was transformed into *Agrobacterium* strain GV3101 using the heat shock method. *Arabidopsis thaliana* Columbia (Col-0) was grown in a greenhouse at 22 °C under a 16 h/8 h light/dark cycle. Transformation was performed using the floral dip method [51]. Positive plants were selected using kanamycin in selection media and verified by polymerase chain reaction (PCR) methods. The transgenic plants in the T_1_ generation with 3:1 positive: negative proportion were regarded as single-copy transgenic plants.

### 2.8. Oil Content and Fatty Acid Composition of Transgenic Arabidopsis

Transgenic Arabidopsis seeds were collected from the T_3_ generation and used for oil content and fatty acid composition analysis. Briefly, approximately 0.1 g of Arabidopsis seeds were ground to powder, and the fatty acid profile was detected using the Agilent 8890 gas chromograph (Agilent, Santa Clara, CA, USA) with C19:0 as the internal standard [52]. The statistically significant differences were determined by Student’s *t*-test. 

### 2.9. RNA Isolation and Real-time Quantitative PCR (RT-qPCR)

Total RNA of the ZM24 upland cotton variety and transgenic Arabidopsis plants was isolated using the RNAprep Pure Plant Kit (TIANGEN, Beijing, China) following the manufacturer’s protocol. After genomic DNA digestion, approximately 1 μg of total RNA was used for reverse transcription using the PrimeScript^TM^ RT reagent Kit (TaKaRa, Dalian, China) according to the manufacturer’s instructions. Specific primers were designed using Primer6 software based on the coding nucleotide sequences of *GhDGAT*, *GhWSD1*, and oil-related genes in Arabidopsis (Table S1). RT-qPCR analysis was performed as described by Zhao et al [25], and the housekeeping cotton genes *GhUBQ7* and Arabidopsis *AtSYL8* were used as internal references. All experiments were performed in triplicates.

## 3. Results

### 3.1. Identification of GhDGAT and GhWSD Family Genes in Gossypium

**Figure 1:**
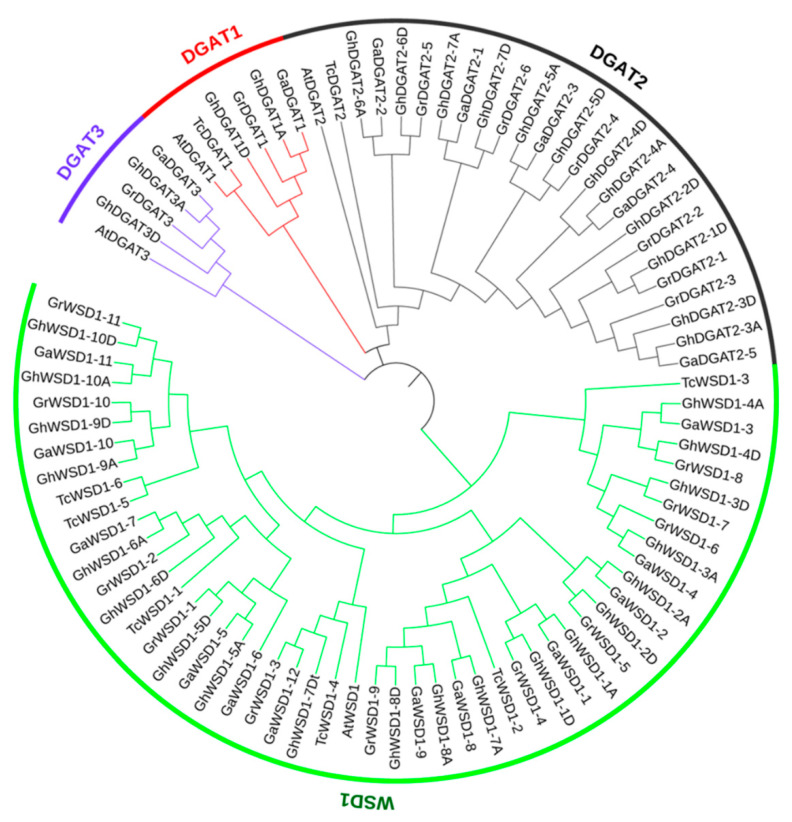
Phylogenetic tree of the DGAT and WSD1 protein family. The phylogenetic tree was constructed using MEGA-X and displayed using iTOL software. At: *Arabidopsis thaliana*; Tc: *Theobroma cacao*; Gh: *G. hirsutum*; Ga: *G. arboretum*; Gr: *G. raimondii*.

Arabidopsis DGAT and WSD amino acid (aa) sequences of AtDGAT1 (AT2G19450), AtDGAT2 (AT3G51520), AtDGAT3 (AT1G48300), and AtWSD1 (AT5G37300) were used as queries for the BLASTp search of the *Gossypium* protein database. The results showed that 19, 19, and 36 *DGAT* and *WSD* genes were detected in the A_2_, D_5_, and AD_1_ genomes, respectively (Table 1 and Table S2). Eight *DGAT* and *WSD* genes were also identified in the cacao plant (*Theobroma cacao*), Malvaceae family. The phylogenetic tree showed that the 86 *DGAT* and *WSD1* genes were classified into four groups: DGAT1, DGAT2, DGAT3, and WSD1 (Figure 1). Moreover, *DGAT* and *WSD* genes in the same group in different cotton genomes tended to form one clade, reflecting the orthologous relationships. The CDS length of the 36 *GhDGAT* and *GhWSD1* genes in *G. hirsutum* ranged from 243 bp (*GhDGAT2-2D*) to 1533 bp (*GhWSD1-6D*). The amino acid sequences of the genes were also characterized. The molecular weight (MW) ranged from 9.06 kDa (*GhDGAT2-2D*) to 57.77 kDa (*GhWSD1-6D*), and the isoelectric point (pI) ranged from 7.66 (*GhWSD1-1A*) to 9.81 (*GhDGAT2-2D*). The subcellular locations of the GhDGAT and GhWSD proteins were predicted by the WoLF PSORT software, and 13 GhDGAT and GhWSD1 proteins were predicted to be located in the cytoplasm (Table 1).

### 3.2. Chromosomal Location and Gene Synteny of GhDGAT and GhWSD1 Genes in G. hirsutum

**Figure 2:**
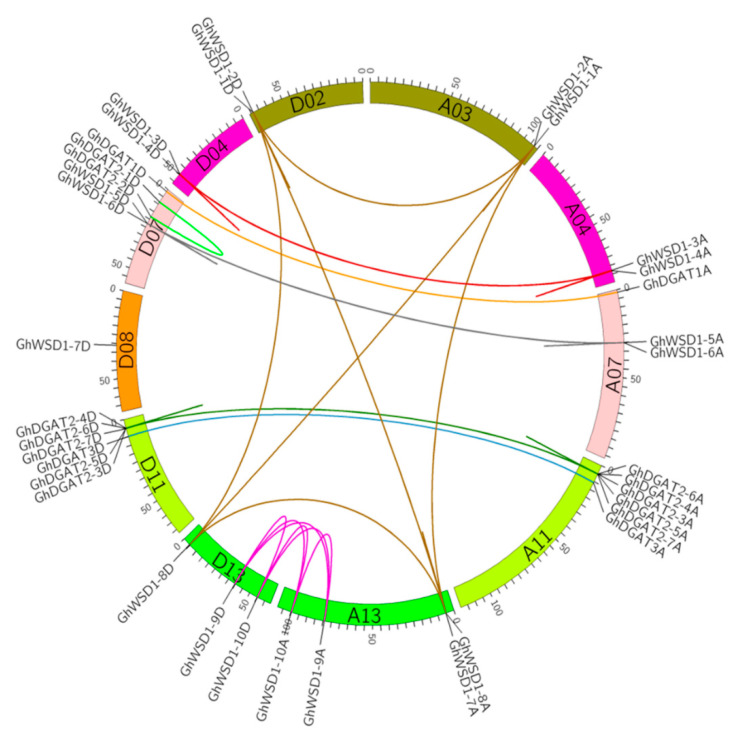
The synteny relationship of *GhDGAT* and *GhWSD1* genes. The relationship was visualized using Circos software. The homologous and homoeologous chromosomes in At and Dt subgenomes are displayed in the same color. The synteny relationship of *GhDGAT* and *GhWSD1* genes are detectable in different colors. Light green lines: paralog genes of *GhDGAT2-1D* and *GhDGAT2-2D*; bottle green lines: duplicate genes of *GhDGAT2*; blue lines: paralog genes of *GhDGAT3*; dark brown lines: ortholog or paralog genes of *GhWSD1-1*, *GhWSD1-2*, *GhWSD1-8*, and *GhWSD1-7A*; red lines: ortholog or paralog genes of *GhWSD1-3* and *GhWSD1-4*; orange lines: paralog genes of *GhDGAT1*; pink lines: ortholog or paralog genes of *GhWSD1-9* and *GhWSD1-10*; grey lines: ortholog or paralog genes of *GhWSD1-5* and *GhWSD1-6*.

To reveal the homologous and homoeologous relationships of the *GhDGAT* and *GhWSD1* genes, gene localization on chromosomes and gene duplication analyses were performed. We observed that most of the GhDGAT and GhWSD1 loci were highly parallel in the At and Dt subgenomes. The *GhDGAT* and *GhWSD1* genes’ number and location on the At subgenomic chromosomes were similar to those on its homoeologous chromosomes in the Dt subgenome (Figure S1). The exception was *GhDGAT2-1D* and *GhDGAT2-2D*, which did not have homologs in the At subgenome, indicating that it might have been lost during evolution. However, the orthologs of GhDGAT2-1D and GhDGAT2-2D were present in *G. raimondii* (*GrDGAT2-1* and *GrDGAT2-2*, respectively). Gene synteny analysis was performed to identify the duplicated genes of *GhDGAT* and *GhWSD1* (Figure 2). The results showed that the cluster of GhDGAT2 might be duplicated genes, which belong to one synteny block. Moreover, *GhWSD1-1A/D*, *GhWSD1-2A/D*, *GhWSD1-7*, and *GhWSD1-8A/D*; GhWSD1-3A/D and GhWSD1-4A/D; GhWSD1-5A/D and GhWSD1-6A/D; and GhWSD1-5A/D and GhWSD1-6A/D might be duplicated genes (Figure 2). The results indicated that the GhDGAT and GhWSD1 genes were frequently duplicated during cotton evolution. According to the location of the duplicated genes, GhDGAT2-3A/D, GhDGAT2-4A/D, GhDGAT2-5A/D, GhDGAT2-6A/D, and GhDGAT2-7A/D; GhWSD1-1A/D and GhWSD1-2A/D; GhWSD1-1A/D and GhWSD1-2A/D; GhWSD1-1A/D and GhWSD1-2A/D; GhWSD1-3A/D and GhWSD1-4A/D; and GhWSD1-5A/D and GhWSD1-6A/D exhibited tandem duplication, whereas GhWSD1-9A/D and GhWSD1-10A/D might have resulted from segmental duplication.

### 3.3. The Conserved Structure and Motifs in GhDGAT and GhWSD1 Proteins

**Figure 3:**
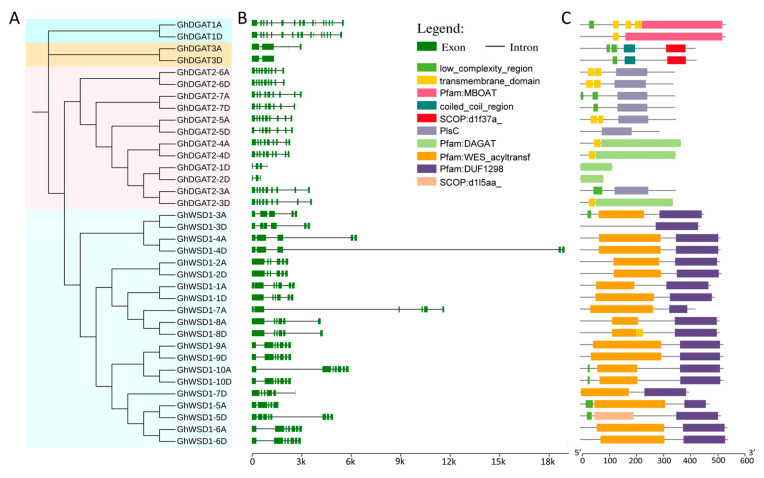
Gene structure and conserved domains in GhDGAT and GhWSD1 members; (**A**) phylogenetic tree of GhDGAT and GhWSD1 proteins; (**B**) gene structure of exons and introns in *GhDGAT* and *GhWSD1* genes; (**C**) the conserved domains in GhDGAT and GhWSD1 proteins.

Upland cotton occupies the largest area of cultivated cotton globally and, therefore, more attention was paid to it during this study. A total of 36 GhDGAT and GhWSD1 proteins were used in the phylogenetic and gene structure analyses. The results showed that the phylogenetic relationships of GhDGAT and GhWSD1 proteins were in accordance with those of other cotton species (Figure 3A). The gene structure analysis showed that exon numbers ranged from 2 to 16. *GhDGAT3D* contains only one intron, most of the *GhDGAT2* genes contain nine exons, whereas *GhDGAT1* homoeologous genes contained 10 exons (Figure 3B). Consistently, GhDGAT and GhWSD1 genes with similar structures were grouped in the same clade.

Furthermore, the conserved domains were detected, GhDGAT1 homologs contained the MBOAT domain, GhDGAT3 homologs contained the SCOP:d1f317a domain; GhDGAT5, GhDGAT6, and GhDGAT7 homologs, and GhDGAT2-3A contained the PlsC domain. GhDGAT2-1D, GhDGAT2-2D, GhDGAT2-3D, and GhDGAT2-4 homologs contained the DAGAT domain. Characteristically, all the GhWSD1 proteins contained the DUF2198 domain, most of which have a WES_acyl transferase domain, except for GhWSD1-3D and GhWSD1-5D. However, only GhDGAT1, GhDGAT2-4, and GhDGAT2-6 homologs and GhDGAT2-3D and GhDGAT2-5A contained a transmembrane domain structure (Figure 3C). Additionally, the top 10 conserved motifs were identified in GhDGAT and GhWSD1 proteins; however, no conserved motifs were detected in GhDGAT1 and GhDGAT3 homologs (Figure S2). The annotation of motifs was in accord with the domains identified in GhDGAT and GhWSD1 proteins (Table S3, Figure 3C).

**Figure 4:**
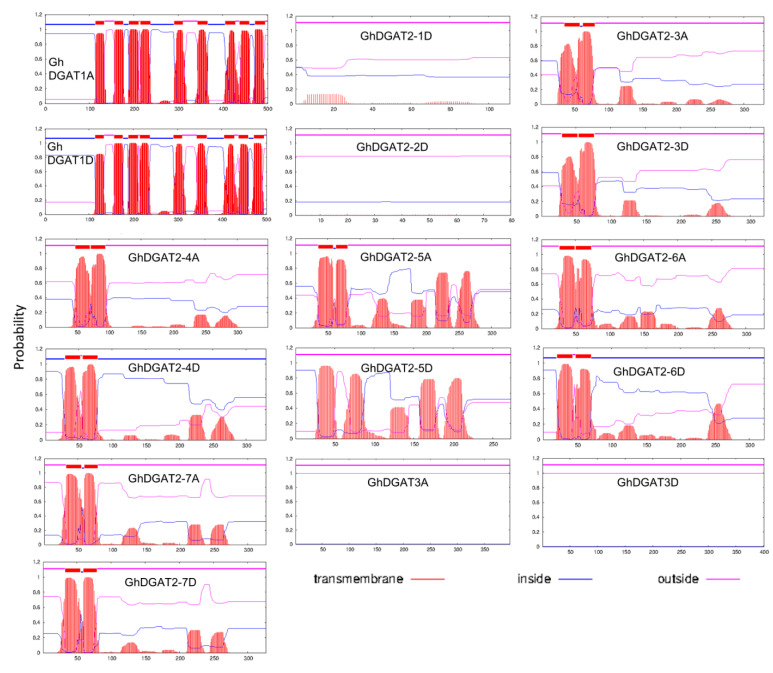
Predicted transmembrane domain for GhDGAT proteins. Regions of GhDGAT amino acid sequences predicted to be located inside or outside the membrane are shown in blue and pink, respectively.

Transmembrane (TM) structures were predicted, and comparisons were made between GhDGAT1, GhDGAT2, GhDGAT3, and GhWSD1 proteins (Figure 4 and Figure S3). GhDGAT1 proteins contain nine TMs, whereas most of the GhDGAT2 proteins contain two TMs, except *GhDGAT2-1D*, *GhDGAT2-2D*, and *GhDGAT2-5D*. *DGAT3* does not have TMs, consistent with its soluble nature. Consistently, only GhDGAT1 proteins had a membrane-bound acyltransferase (MBOAT) domain, whereas GhDGAT2, GhDGAT3, and GhWSD1 did not have an MBOAT domain (Figure 4 and Figure S3). These results support the hypothesis that DGAT1 and DGAT2 belong to different gene families and evolved separately during eukaryote evolution, as demonstrated by the phylogenetic tree (Figure 1).

### 3.4. Cis-Elements in the GhDGAT and GhWSD1 Promoters

**Figure 5:**
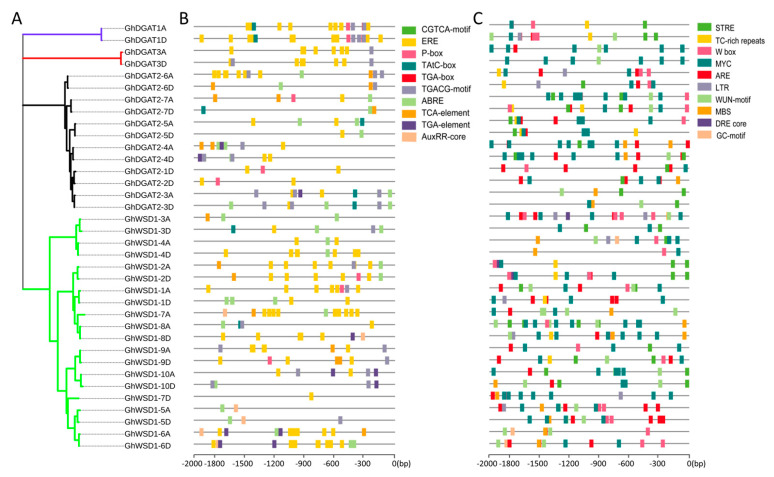
*cis*-elements in GhDGAT and GhWSD1 promoter regions; (**A**) phylogenetic tree of GhDGAT and GhWSD1 proteins; (**B**) predicted cis-elements involved in plant hormones. ABRE: cis-acting element involved in abscisic acid responsiveness; AuxRR-core: cis-acting regulatory element involved in auxin responsiveness; AuxRE: part of an auxin-responsive element; CGTCA-motif: cis-acting regulatory element involved in MeJA-responsiveness; GARE-motif: gibberellin-responsive element; TGACG-motif: cis-acting regulatory element involved in MeJA-responsiveness; TGA-element: auxin-responsive element; TGA-box: part of auxin-responsive element; ERE: cis-acting ethylene responsive element; P-box: gibberellin-responsive element; (**C**) predicted cis-elements involved in environmental stress responses. GC-motif: enhancer-like element involved in anoxic specific inducibility; LTR: cis-acting element involved in low-temperature responsiveness; MBS: MYB binding site involved in drought-inducibility; STRE: stress response element; TC-rich repeats: cis-acting element involved in defense and stress responsiveness; WUN-motif: wound-responsive element; MYC: cis-acting element involved in drought stress; W box: cis-acting element involved in sugar metabolism and plant defense signaling; DRE core: dehydration-responsive element; ARE: cis-acting regulatory element essential for anaerobic induction.

The 2 kb sequence upstream of the start codon (ATG) of *GhDGAT* and *GhWSD1* genes was used to investigate the cis-elements in the promoter regions in the PlantCARE database. A total of 93 cis-elements were predicted in the promoter regions of the 36 *GhDGAT* and *GhWSD1* genes. Several cis-elements were implicated in light response. Moreover, the roles of cis-elements in environmental stresses and hormone responses are highlighted in Figure 5. Among the predicted hormone response elements, ERE, ABRE, and CGTCA-motif were the most abundant, indicating that *GhDGAT* and *GhWSD1* genes may primarily respond to ethylene, abscisic acid, and MeJA (Figure 5B). Ten environmental stress-related elements were identified, with most of them involving drought stress (MYC), stress response (STRE), and anaerobic induction (ARE) (Figure 5C). 

### 3.5. Target Transcription Factors and miRNAs of GhDGAT and GhWSD Genes

Transcription factors (TFs) regulate the precise initiation of gene transcription. Therefore, we identified the target TFs of GhDGAT and GhWSD1 using the PlantRegMap server, and a total of 568 relationships were identified (Figure S4). GhWSD1-1D may be regulated by more TFs, such as the stress TFs ethylene response factor (ERF), DNA binding with one finger (Dof), or v-myb avian myeloblastosis viral oncogene homolog (MYB). Moreover, several *GhDGAT* and *GhWSD1* genes were regulated by Dof, ERF, and NAC (NAM, ATAF, and CUC) TFs, indicating that these TFs regulate plant development and stress responses.

**Figure 6:**
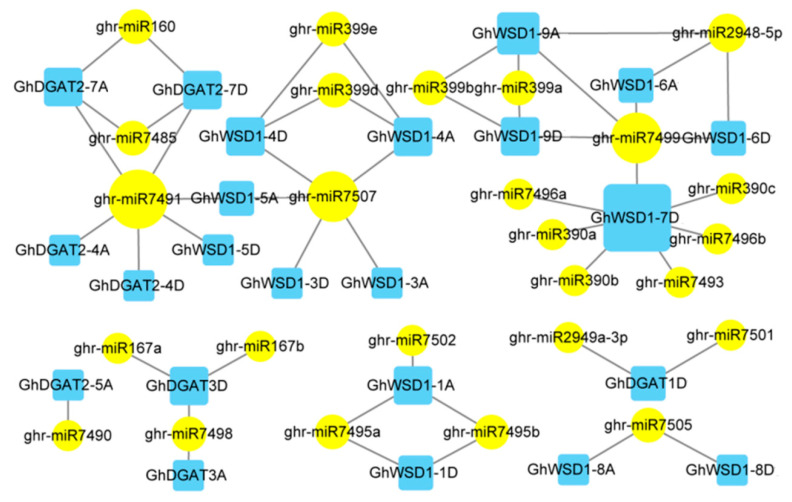
miRNA targets *GhDGAT* and *GhWSD1* genes. The predicted regulation miRNAs are marked with a round yellow background, the target *GhDGAT* and *GhWSD1* genes are marked with blue rectangles. The regulation and targeting levels are shown with varying degrees.

miRNAs have been widely studied in the regulation of gene expression, which plays an important role in abiotic stress responses. To explore the potential role of miRNAs in regulating *GhDGAT* and *GhWSD1* genes, 26 putative miRNAs targeting 23 *GhDGAT* and *GhWSD1* genes were predicted using the psRNATarget website, including 52 interaction relationships (Figure 6). We observed that GhWSD1-7Dt was the most targeted, interacting with seven miRNAs. ghr-miR71491 was the most regulated miRNA, and was involved in regulating four *GhDGAT* and *GhWSD1* genes. Additionally, we observed that most *GhDGAT* and *GhWSD1* homologs were regulated by the same miRNAs, suggesting similar functional roles.

### 3.6. Gene Expression Profile of Upland Cotton in Response to Abiotic Stresses 

**Figure 7:**
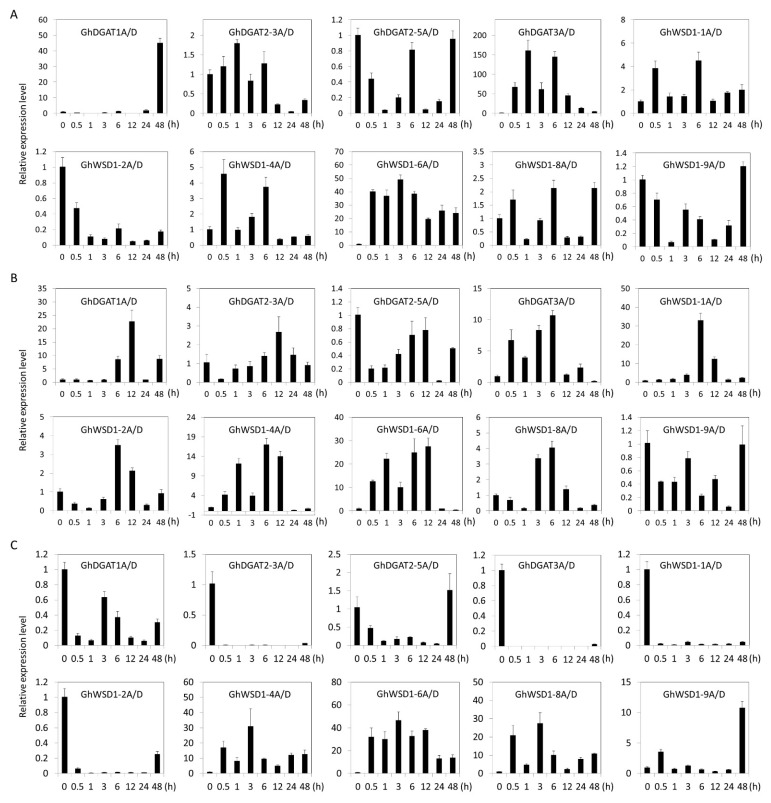
Expression profiles of *GhDGAT* and *GhWSD1* genes during abiotic stresses; (**A**) gene expression patterns under cold stress; (**B**) gene expression patterns under drought stress; (**C**) gene expression patterns under salt stress. Cold: 4 °C; drought: 17% PEG-6000; salt: 200 mM NaCl.

Furthermore, the expression profiles of the *GhDGAT* and *GhWSD1* genes under abiotic stresses, including cold, drought, and salt stress were investigated at different time series (Figure 7). The *GhDGAT* or *GhWSD1* genes that were not expressed in cotton leaves were unaffected. The results showed that there were differences in the expression profiles of the *GhDGAT1*, *GhDGAT2*, *GhDGAT3*, and *GhWSD1-1* genes under the different abiotic stress conditions. 

The expression of *GhDGAT1* was upregulated at 48 h under cold stress. Additionally, the expression level of *GhDGAT3A/D* and *GhWSD1-6A/D* increased at several time points under cold stress (Figure 7A). However, most *GhDGAT* and *GhWSD1* genes were upregulated under drought stress, except for *GhDGAT2-5A/D* and *GhWSD1-9A/D*, indicating that *GhDGAT* and *GhWSD1* genes respond to drought stress (Figure 7B). The expression of *GhDGAT1A/D*, *GhDGAT2-3A/D*, *GhDGAT2-5A/D*, *GhDGAT3A/D*, *GhWSD1-1A/D*, and *GhWSD1-2A/D* was downregulated, and that of *GhWSD1-4A/D*, *GhWSD1-6A/D*, *GhWSD1-8A/D*, and *GhWSD1-9A/D* was upregulated under salt stress (Figure 7C). The expression profiles of *GhDGAT* and *GhWSD1* genes under abiotic stress acted in cooperation with many environment response elements that were predicted in their promoter regions (Figure 5).

### 3.7. Expression Profiling of DGAT and WSD1 Genes in Cotton Development

Gene expression models are important for gene function analysis. The gene expression patterns of *DGAT* and *WSD1* in developing cottonseeds of *G. arboretum*, *G. raimondii*, and *G. hirsutum* were investigated [45]. DGAT1 genes showed increased expression levels at later developmental stages of 30 and 40 days post anthesis (DPA). Compared with the higher expression levels of DGAT1 genes in *G. arboretum* and *G. raimondii*, there was an impaired expression of *GhDGAT1* in *G. hirsutum*. Additionally, most DGAT2 genes exhibited low expression levels; however, *GrDGAT2-3*, *GhDGAT2-3A/D*, *GaDGAT2-1*, *GhDGAT2-7D*, and *GrDGAT2-6* exhibited high expression levels (Figure S5A). Compared with *DGAT1* and *DGAT2*, *DGAT3* genes showed a more abundant expression in the diploid and tetraploid cotton species. For the WSD1 group, there was an increase in the expression profiles of *GaWSD1-2*, *GhWSD1-1a*, *GrWSD1-4*, *GaWSD1-5*, and *GrWSD1-1*, at 30 and 40 DPA, whereas only GrWSD1-2 was highly expressed at 10 and 20 DPA (Figure S5B). Similar expression patterns were observed in the same groups, and the different expression profiles in diploid and tetraploid cotton species indicated the evolution and differentiation of DGAT and WSD1 proteins.

**Figure 8:**
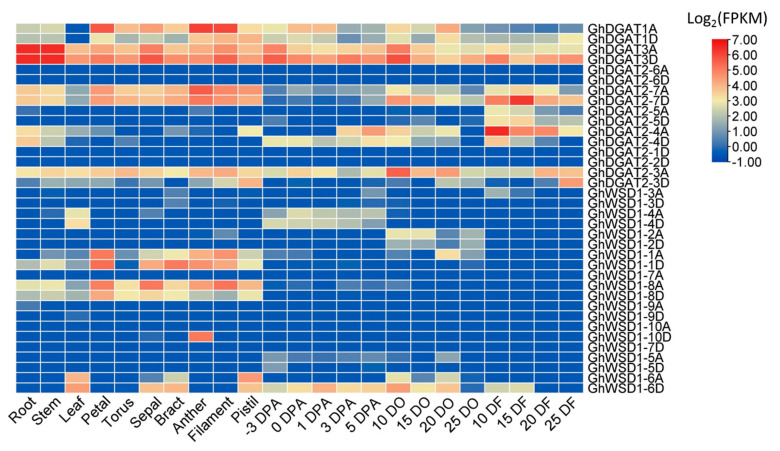
Expression patterns of *GhDGAT* and *GhWSD1* genes in upland cotton. DF: DPA fiber; DO: DPA ovule. Gene expression levels (FPKM) were averaged and normalized by a log2 scale.

Public expression datasets of upland cotton were used for gene expression analyses in different tissues and ovules at different fiber development stages [46]. The expression patterns of *GhDGAT* and *GhWSD1* genes in developing ovules were consistent with those in developing cottonseed (Figure 8 and Figure S5). Several *GhDGAT* and *GhWSD1* genes, including *GhDGAT2-6*, *GhDGAT2-1*, *GhWSD1-3*, *GhWSD1-5*, and *GhWSD1-9* were barely expressed in upland cotton. *GhDGAT1* and *GhWSD1-10D* genes were highly expressed in male reproductive organs (anthers and filaments). The *GhDGAT2-4* gene was highly expressed in ovules during the early development stage (3 and 5 DO) and fiber rapid elongation stage (10 and 15 DF), indicating that it was involved in fiber elongation. *GhDGAT2-7* was highly expressed in reproductive organs (torus, bract, and pistil) and fiber. However, *GhWSD1-1* and *GhWSD1-8* were preferentially expressed in reproductive organs. However, we highlight that *GhDGAT3* genes were consistently and abundantly expressed in cotton development, and *GhDGAT3D* showed a higher expression level than that of *GhDGAT3A* in the developing cottonseed (Figure 8).

### 3.8. Overexpression of GhDGAT3D Improves Oil Content in Arabidopsis Seeds

**Figure 9:**
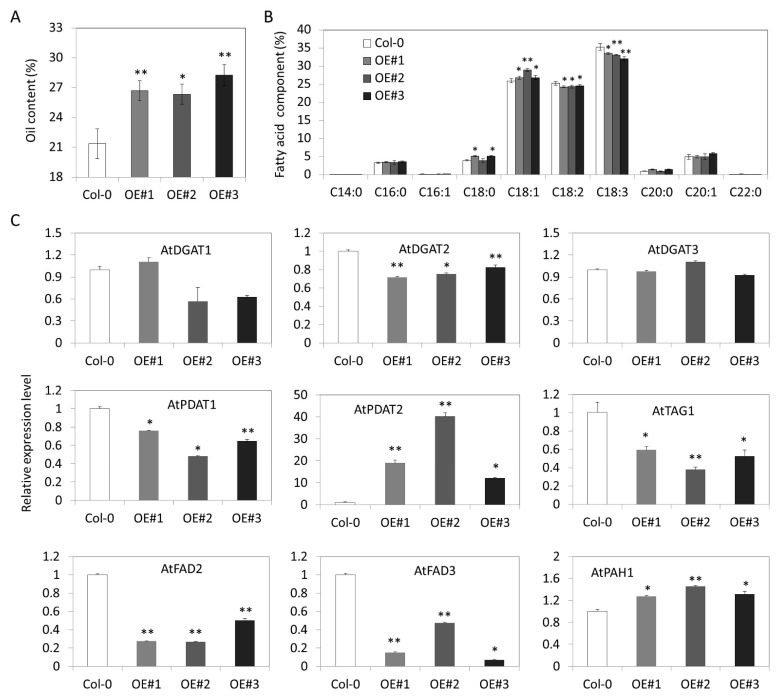
Overexpression of *GhDGAT3D* increased oil content in Arabidopsis seeds; (**A**) total oil content in *GhDGAT3D* overexpressing Arabidopsis seeds; (**B**) fatty acid component in transgenic and the control Arabidopsis seed; (**C**) detection of oil biosynthesis related genes in transgenic Arabidopsis. * and ** indicate data were significantly different at *p*-value of 0.05 and 0.01 levels, respectively.

The recombination vector p35S::*GhDGAT3D* was transformed into Arabidopsis using the floral dip method. Several single transformation events were obtained, among which three single-copy transgenic lines overexpressing *GhDGAT3D* at the mRNA level were selected for further analysis (Figure S6). To confirm the role of *GhDGAT3D* in oil biosynthesis, the oil content and fatty acid composition of the homozygous T_3_ generation of Arabidopsis overexpressing *GhDGAT3D* was determined by gas chromatography. Total oil content was increased to 26.72%, 26.35%, and 28.25% in OE#1, OE#2, and OE#3 transgenic lines, respectively, compared with that of 21.37% in the control plant (Figure 9A). Additionally, there was an increase in C18:1 (26.82%, 28.97%, and 26.84% in OE#1, OE#2, and OE#3 transgenic lines, respectively, compared with that of 25.83% in the control) content and a decrease in C18:2 (24.30%, 24.33%, and 24.60%, compared with that of 25.42%) and C18:3 (33.50%, 33.03%, and 32.04%, compared with that of 35.25%) contents in Arabidopsis seeds (Figure 9B), indicating that *GhDGAT3D* was involved in regulating oil biosynthesis and fatty acid composition of cotton seeds.

The expression levels of oil-related genes were investigated in transgenic Arabidopsis, including *AtDGAT1*, *AtDGAT2*, *AtDGAT3*, *AtPDAT1*, *AtPDAT2*, *AtTAG1*, *AtFAD2*, *AtFAD3*, and *AtPAH2* (Figure 9C). Ectopic expression of *GhDGAT3D* did not affect the expression level of AtDGAT3, indicating that the alteration of the oil content and fatty acid composition of transgenic Arabidopsis resulted from the overexpression of *GhDGAT3D*. Additionally, there was a decrease in the expression levels of *AtDGAT2*, *AtPDAT1*, *AtTAG1*, *AtFAD2*, and *AtFAD3*, and an increase in the expression of *AtPAH* in *GhDGAT3D* overexpressing plants. The results indicated that the increase in C18:1 content and the decrease in C18:2 and C18:3 contents may result from the decrease in the expression levels of *AtFAD2* and *AtFAD3* in transgenic Arabidopsis. However, there was a significant increase in the expression of *AtPDAT2* in *GhDGAT3D* overexpressing Arabidopsis, indicating a potential interaction relationship between DGAT3 and PDAT2 in plant seeds.

## 4. Discussion

### 4.1. Gene Duplication and Functional Diversification of GhDGAT and GhWSD1 Genes

The phylogenetic tree, transmembrane domain and expression analysis showed that the *DGAT1*, *DGAT2*, *DGAT3* and *WSD1* genes showed apparent differences (Figure 1, Figure 4, and Figure 8). These results indicate that they are divergent genes and may have a distinct origin, consistent with what is described in soybeans [53]. Gene duplication partakes a major role in the evolution of plant genomes. The results of the present study showed that *GhDGAT* and *GhWSD1* genes were frequently duplicated during cotton evolution, with only one pair in each of the *GhDGAT1* and *GhDGAT3* genes, and over five pairs of *GhDGAT2* homologs genes identified in upland cotton. It has been reported that there are two paralogs of *DGAT2* genes in maize and five paralogs in soybean (*Glycine max*) [28,30]. Although, there is a close relationship between cacao and cotton, only one *TcDGAT2* gene was found in cacao, whereas five paralogs were found in diploid cottons (*G. arboretum* and *G. raimondii*) (Figure 1), indicating that the duplication of *GhDGAT2* genes occurred after the cotton genus separated from cacao. The multiple paralogs of *GhDAGT2* in soybean and oilseeds, including cotton, confirmed the role of the genes in oil biosynthesis. The cluster of *GhDGAT2* genes in the chromosomes of upland cotton showed that tandem duplication events occurred during cotton evolution. Gene duplication events also occurred in *GhWSD1* genes, with only one *AtWSD1* gene in Arabidopsis, six *TcWSD1* genes in cacao, and over 10 *WSD1* genes detected in diploid cottons (*G. arboretum* and *G. raimondii*). Additionally, tandem duplication events occurred in *GhWSD1* genes, among which *GhWSD1-1A/D*, *GhWSD1-2A/D*, *GhWSD1-3A/D* and *GhWSD1-4A/D*, *GhWSD1-5A/D*, and *GhWSD1-6A/D* showed tandem duplication in upland cotton (Figure 2). Overall, the results showed that *GhWSD1* genes were frequently duplicated before and after cotton division from cacao.

*DGAT* genes have been reported to be involved in TAG biosynthesis and abiotic stress responses [8,17,54]. Therefore, the expression profiles of *GhDGAT* and *GhWSD1* genes were investigated in the present study. The results showed that several paralogs of *GhDGAT2* genes were barely expressed in cotton, except *GhDGAT2-7A/D*, indicating that *GhDGAT2* genes may have experienced functional diversification or shown gene redundancy during cotton evolution. *GhWSD1* genes showed intricate expression patterns during cotton developmental stages and under abiotic stresses. Specifically, *GhWSD1-1A/D* responded to cold and drought stresses at several time points; however, the duplicate genes of *GhWSD1-2A/D* did not respond to any stress condition. The diverse expression patterns indicated that *GhWSD1* genes also experienced functional diversification.

### 4.2. GhDGATs and GhWSD1s Response to Abiotic Stresses

DGAT1 appears to play a role in freezing and drought stress responses in Arabidopsis [54], *Brassica napus* [55] and *Boechera stricta* [56]. *BsDGAT1* was higher expression in freeze-tolerant plants than freeze-susceptible plants; overexpression of *AtDGAT1* increased freezing tolerance in Arabidopsis [56], whereas Arabidopsis *DGAT1* (*AtDGAT1*) defective mutant lines were sensitive to freezing [54]. Additionally, overexpression of *DGAT1* in *B. napus* is shown to reduce the negative effects of drought on seed oil content [55]. In the present study, there was an increase in the expression of *GhDGAT1A/D* homologs in Arabidopsis at 48 h under cold conditions. Additionally, there was an increase in the expression of *GhDGAT1* at several time points under drought stress, indicating that *GhDGAT1* was involved in cold and drought stress responses. The role of DGAT2 in abiotic stress remains unclear; however, the results of the present study showed that there was an increase in the expression of *GhDGAT2-3A/D* under cold and drought stresses, but it decreased invariably under salt stress (Figure 7). Additionally, there was a decreasing trend in the expression of *GhDGAT2-5A/D* in response to drought and salt stress conditions. The inconsistent expression patterns of *GhDGAT2* in response to different environmental stresses indicated the complicated role of *GhDGAT2* in environmental adaptation. The function of DGAT3 in abiotic stress is scarcely reported. *GhDGAT3* genes were highly expressed in the root, stem, and other tissues. Meanwhile, there was an increase in the expression of *GhDGAT3* genes under cold and drought stress, whereas the expression of the gene was reduced under salt stress conditions (Figure 7). Moreover, several MYC cis-elements were found in the GhDGAT3A/D promoter region, as MYC is a cis-acting element involved in drought stress (Figure 5C). These results indicate that GhDGAT3 may be involved in cold and drought stress responses in cotton.

Patwari et al. (2019) reported that WSD1 responds to drought stress [26]. *WSD1* is upregulated in grape berries in response to drought [57]. An R2R3-type MYB94 transcription factor activates the Arabidopsis cuticular wax biosynthesis gene *WSD1* and may be important in the plant response to drought stress [58]. Another AP2/ERF-type transcription factor, WRINKLED4, binding the WSD1 promoter specifically, controls cuticular wax biosynthesis [59]. In the present study, *GhWSD1-1* genes were highly expressed under drought stress, confirming the function of GhWSD1 in the drought response (Figure 7B). Additionally, there was an increase in the expression profiles of *GhWSD1-4A/D*, *GhWSD1-6A/D*, *GhWSD1-8A/D*, and *GhWSD1-9A/D* under cold and salt stress at several time points, indicating the multiple roles of the *GhWSD1* genes in the spread of upland cotton to different regions (Figure 7A,C).

### 4.3. Role of GhDGATs in Oil Biosynthesis Regulation

DGAT1 has been functionally confirmed in oil biosynthesis in Arabidopsis, soybean, and oilseed rape [17]. Expression analysis revealed that *DGAT1* was abundantly expressed in developing embryos in several oilseed crops and its transcript level according to oil accumulation in developing seed [60]. In the present study, there was an increase in the expression of *GhDGAT1* in cotton seeds at 10 and 20 DO (Figure 8), which corresponded with the rapid oil accumulation stage in cottonseed, indicating that *GhDGAT1* was important in TAG biosynthesis. Moreover, there were high expression levels of *GhDGAT1* in the petals, anthers, and filaments of upland cotton, indicating that *GhDGAT1* might be involved in the reproductive development of upland cotton. It was reported that TAG production via DGAT1 and DGAT2 occurs in a distinct ER subdomain; moreover, it has been reported that tung tree DGAT1 and DGAT2 proteins are localized to different ER regions and differ in substrate preference [61]. The expression level of *DGAT2* was found to be higher in unusual or polyunsaturated fatty acids accumulating in developing seeds. *Cyperus esculentus* CeDGAT2b has been shown to have a substrate preference for UFA for TAG synthesis [62]. Ectopic overexpression of *CeDGAT2* has been shown in enhanced oil and C18:1 accumulation in tobacco leaves [63]. In the present study, only *GhDGAT2-3A* and *GhDGAT2-7D* exhibited high expression levels during cottonseed development, whereas the other 10 paralogs of *GhDAGT2* genes were barely expressed. However, we found that *GhDGAT2-7A* and *GhDGAT2-4D* were abundantly expressed at 10 to 20 DPA during fiber development, indicating that the two genes may be involved in fiber elongation. 

Few studies have focused on the role of DGAT3 in TAG biosynthesis. To date, only AtDGAT3 and CsDGAT3 are confirmed as metalloproteins involved in TAG biosynthesis in plants [23,24]. The DGAT3 protein has not been identified in mossy or algal species [28], indicating that it may have evolved during plant evolution. *GhDGAT3* is regarded as a key candidate gene for the total triglyceride pool [64]. *GmDGAT3* has the highest transcript levels when compared to other *GmDGAT* genes in developing soybean seeds, suggesting that GmDGAT3 is probably involved in TAG synthesis [53]. The expression level of *GhDGAT3* is significantly higher than that of *GhDGAT1* and *GhDGAT2* during cottonseed development [3]. In the present study, we observed that *GhDGAT3* was highly expressed in the ovule of upland cotton and during fiber development (Figure 8). Moreover, there was a significant increase in the oil content of *GhDGAT3D* overexpressing Arabidopsis transgenic plants compared with that of the control plants, indicating that *GhDGAT3* was involved in oil biosynthesis. Additionally, there was a decrease in the C18:2 and C18:3 contents and an increase in the C18:1 content of the seeds of *GhDGAT3D*, overexpressing Arabidopsis transgenic plants (Figure 9B). These results are consistent with the transcript levels of *AtFAD2* and *AtFAD3* being weakened in transgenic Arabidopsis (Figure 9C). In the present study, the expression assay results showed that most *GhWSD1* genes were barely or not expressed in developing cottonseed. Moreover, it has been reported that the WSD1 enzyme shows deficient levels of diacylglycerol acyltransferase activity [25]. However, few studies have confirmed the role of WSD1 in oil biosynthesis. Overall, the WSD1 enzyme may be more involved in environmental stress responses than in oil biosynthesis.

## 5. Conclusions

In summary, GhDGATs and GhWSD1s were identified and classified in upland cotton; additionally, their roles in stress responses, oil biosynthesis, and fatty acid composition were also elucidated. The findings of this study showed that WSD1 genes were mostly involved in stress responses, whereas DGAT genes were involved in both oil synthesis, fatty acid composition, and abiotic stress responses. Overall, the findings of this study contribute to the understanding of DGAT and WSD1 genes in fatty acid biosynthesis and abiotic stress responses in cotton.

## Figures and Tables

**Table 1:** Characterization of *GhDGAT* and *GhWSD1* genes in *G. hirsutum*.^a^ The top three most possible subcellular localizations of *GhDGAT* and *GhWSD1* genes are shown. Abbreviations: chlo: chloroplast; cysk: cytoskeleton; cyto: cytoplasm; E.R.: endoplasmic reticulum; extr: extracellular; mito: mitochondrion; nucl: nucleus; pero: peroxisome; plas: plastid; vacu: vacuole.

Name	Gene Locus ID	Nucleic acid			Amino Acid				
		Location	CDS	Exons	Size	Mw (Da)	pI	Formula	Subcellular Location ^a^
GhDGAT1A	Gh_A07G012600	A07:1460679-1467181	1509	16	502	57,879.75	9.21	C_2653_H_4088_N_696_O_697_S_31_	plas: 8, E.R.: 3, vacu: 2
GhDGAT1D	Gh_D07G014100	D07:1401161-1407228	1509	16	502	57,845.69	9.15	C_2657_H_4078_N_692_O_696_S_31_	plas: 8, E.R.: 3, vacu: 2
GhDGAT2-1D	Gh_D07G075800.1	D07:8705721-8706732	336	4	111	12,493.81	9.66	C_574_H_901_N_151_O_147_S_7_	extr: 8, chlo: 3, cyto: 3
GhDGAT2-2D	Gh_D07G142000.1	D07:21465697-21466248	243	3	80	9063.68	9.81	C_415_H_647_N_111_O_107_S_5_	chlo: 8.5, chlo_mito: 6
GhDGAT2-3A	Gh_A11G060400	A11:5266893-5270919	993	9	330	37,355.86	9.55	C_1751_H_2664_N_446_O_438_S_12_	E.R.: 4, chlo: 3, plas: 2
GhDGAT2-4A	Gh_A11G060500	A11:5273930-5276602	1047	9	348	39,547.3	8.92	C_1835_H_2791_N_471_O_470_S_18_	cyto: 6, E.R.: 4, plas: 2
GhDGAT2-5A	Gh_A11G060600	A11:5277328-5279908	993	9	330	37,828.59	9.07	C_1779_H_2692_N_440_O_442_S_16_	plas: 10, E.R.: 3, cyto: 1
GhDGAT2-6A	Gh_A11G060700	A11:5282801-5284857	984	9	327	37,172.99	9.31	C_1767_H_2651_N_427_O_426_S_15_	E.R.: 4, cyto: 3, mito: 2
GhDGAT2-7A	Gh_A11G060800	A11:5292926-5296526	984	9	327	37,216.82	9.02	C_1759_H_2643_N_433_O_432_S_14_	plas: 9, cyto: 2, E.R.: 2
GhDGAT2-3D	Gh_D11G060500	D11:5049906-5053735	963	10	320	36,441.79	9.62	C_1706_H_2597_N_439_O_425_S_12_	chlo: 4, E.R.: 3, plas: 2
GhDGAT2-4D	Gh_D11G060600	D11:5056982-5059729	993	9	330	37,703.24	9.12	C_1762_H_2673_N_447_O_444_S_15_	plas: 6, cyto: 5, E.R.: 2
GhDGAT2-5D	Gh_D11G060700	D11:5060331-5062945	822	8	273	31,100.5	9.06	C_1455_H_2204_N_364_O_368_S_13_	E.R.: 4, chlo: 3, cyto: 2
GhDGAT2-6D	Gh_D11G060800	D11:5065409-5067493	969	9	322	36,800.52	9.47	C_1745_H_2627_N_429_O_421_S_14_	cyto: 7, E.R.: 5, chlo: 1
GhDGAT2-7D	Gh_D11G060900	D11:5079608-5083224	984	8	327	37,337.91	9.13	C_1761_H_2650_N_436_O_435_S_14_	plas: 7, E.R.: 4, cyto: 2
GhDGAT3A	Gh_A11G111700	A11:10691786-10694445	1197	4	398	43,064.36	8.83	C_1836_H_3089_N_549_O_594_S_22_	chlo: 13, cyto: 1
GhDGAT3D	Gh_D11G112600	D11:9900476-9902133	1206	2	401	43,483.87	8.72	C_1853_H_3114_N_550_O_601_S_24_	chlo: 11, nucl: 2, plas: 1
GhWSD1-1A	Gh_A03G216700	A03:107525052-107527779	1359	7	452	50,570.87	7.66	C_2268_H_3628_N_606_O_651_S_24_	pero: 6, E.R.: 3, plas: 2
GhWSD1-2A	Gh_A03G216900	A03:107537659-107540301	1449	6	482	54,196.22	9.13	C_2439_H_3871_N_663_O_678_S_27_	pero: 6, E.R.: 3, plas: 2
GhWSD1-3A	Gh_A04G123200	A04:77942098-77944989	1248	5	427	47,674.48	9.2	C_2152_H_3429_N_569_O_612_S_19_	cyto: 4, cysk: 4, chlo: 3
GhWSD1-4A	Gh_A04G123300	A04:78055366-78062208	1455	5	484	53,607.04	8.79	C_2418_H_3835_N_629_O_703_S_20_	cyto: 7, vacu: 3, E.R.: 3
GhWSD1-5A	Gh_A07G148000	A07:30093118-30095490	1341	5	446	50,742.54	9.15	C_2277_H_3624_N_620_O_659_S_16_	cyto: 12, nucl: 1, cysk: 1
GhWSD1-6A	Gh_A07G148100	A07:30196177-30199726	1527	7	508	57,493.34	8.61	C_2569_H_4085_N_703_O_746_S_23_	nucl: 5, cyto: 5, chlo: 3
GhWSD1-7A	Gh_A13G041500	A13:5011174-5023459	1197	6	398	44,858.97	8.82	C_1998_H_3213_N_567_O_571_S_17_	cyto: 7, E.R.: 6, chlo: 1
GhWSD1-8A	Gh_A13G041600	A13:5057176-5061568	1446	6	481	53,683.46	8.01	C_2409_H_3851_N_641_O_693_S_25_	pero: 6, E.R.: 3, plas: 2
GhWSD1-9A	Gh_A13G131300	A13:79197177-79199666	1485	7	494	56,054.04	9	C_2536_H_3995_N_667_O_718_S_23_	cyto: 8, nucl: 2, E.R.: 2
GhWSD1-10A	Gh_A13G184900	A13:99672162-99678336	1488	7	495	56,441.83	9.12	C_2556_H_4040_N_672_O_714_S_26_	cyto: 8, E.R.: 3, vacu: 2
GhWSD1-1D	Gh_D02G233400	D02:68707959-68711014	1395	6	464	52,008.54	8.32	C_2322_H_3724_N_628_O_669_S_27_	cyto: 5, E.R.: 4, vacu: 2
GhWSD1-2D	Gh_D02G233600	D02:68719514-68721802	1467	6	488	54,607.78	8.95	C_2460_H_3904_N_664_O_683_S_28_	pero: 11, nucl: 2, cyto: 1
GhWSD1-3D	Gh_D04G163100	D04:49567089-49570803	1245	6	414	46,512.18	9.09	C_2112_H_3350_N_550_O_595_S_17_	cyto: 4, golg: 3, vacu: 2
GhWSD1-4D	Gh_D04G163400	D04:49689723-49709898	1455	5	484	53,537.8	8.73	C_2413_H_3816_N_632_O_703_S_19_	cyto: 8, chlo: 3, nucl: 1
GhWSD1-5D	Gh_D07G148000	D07:22700357-22705541	1458	8	485	55,118.19	6.96	C_2485_H_3905_N_671_O_720_S_13_	chlo: 3, plas: 3, cyto: 2
GhWSD1-6D	Gh_D07G148100	D07:22756404-22759955	1533	7	510	57,768.58	8.48	C_2576_H_4094_N_708_O_751_S_24_	nucl: 5, cyto: 5, chlo: 2
GhWSD1-7D	Gh_D08G104800	D08:31044870-31047654	1131	6	376	42,022.47	9.53	C_1907_H_3079_N_507_O_525_S_16_	chlo: 4, cyto: 3, nucl: 2.5
GhWSD1-8D	Gh_D13G043400	D13:4582655-4587177	1446	6	481	53,776.75	8.59	C_2415_H_3862_N_644_O_687_S_27_	pero: 6, E.R.: 3, plas: 2
GhWSD1-9D	Gh_D13G132800	D13:39715595-39718096	1485	7	494	56,210.27	9	C_2550_H_4003_N_669_O_715_S_23_	cyto: 6, E.R.: 4, nucl: 2
GhWSD1-10D	Gh_D13G187700	D13:55277000-55279722	1488	7	495	56,200.28	8.99	C_2543_H_4009_N_667_O_719_S_24_	cyto: 7, E.R.: 3, vacu: 2

## Data Availability

The data presented in this study are available in Supplementary Materials.

## Supplementary Materials

The following are available online at https://www.mdpi.com/article/10.3390/genes12071045/s1, Table S1. Primers used in this study. Table S2. Identification and nomenclature of *DGAT* and *WSD1* genes in *G. arboreum* and *G. raimondii*. Table S3. Annotation of the conserved motifs identified in GhDGAT and GhWSD1. Figure S1. Location of *GhDGAT* and *GhWSD1* genes in the upland cotton genome. Figure S2. Conserved motifs identified in GhDGAT and GhWSD1 proteins; (A) phylogenetic tree of GhDGAT and GhWSD1 proteins; (B) top 10 conserved motifs; (C) logos of the ten conserved motifs in GhDGAT and GhWSD1 proteins. Figure S3. Predicted transmembrane domain of GhWSD1 proteins. Regions of GhWSD1 amino acid sequences predicted to be located inside or outside the membrane are shown in blue and pink, respectively. Figure S4. Predicted target TFs of *GhDGAT* and *GhWSD1* genes. The predicted regulation miRNAs are depicted as round in yellow background, the target *GhDGAT* and *GhWSD1* genes as rectangles in blue background. Figure S5. Expression patterns of *GhDGAT* and *GhWSD1* genes in developing cottonseed; (A) transcript levels of *GhDGAT* genes in developing cottonseed; (B) expression profiles of *GhWSD1* genes in cottonseed development. DPA: days post anthesis. Gene expression levels (FPKM) were averaged and normalized by log_2_ scale. Figure S6. Identification of the positive transgenic Arabidopsis in RNA level. *AtSYL8*: the internal reference; OE#1, OE#2, OE#3: transgenic Arabidopsis lines.
