# Supplementary figures and images for: Evolution and Characterization of Acetyl Coenzyme A: Diacylglycerol Acyltransferase Genes in Cotton Identify the Roles of GhDGAT3D in Oil Biosynthesis and Fatty Acid Composition
Source: Genes (Basel). 2021 Jul 7;12(7):1045. doi: 10.3390/genes12071045 (PMC8306077; doi:10.3390/genes12071045)

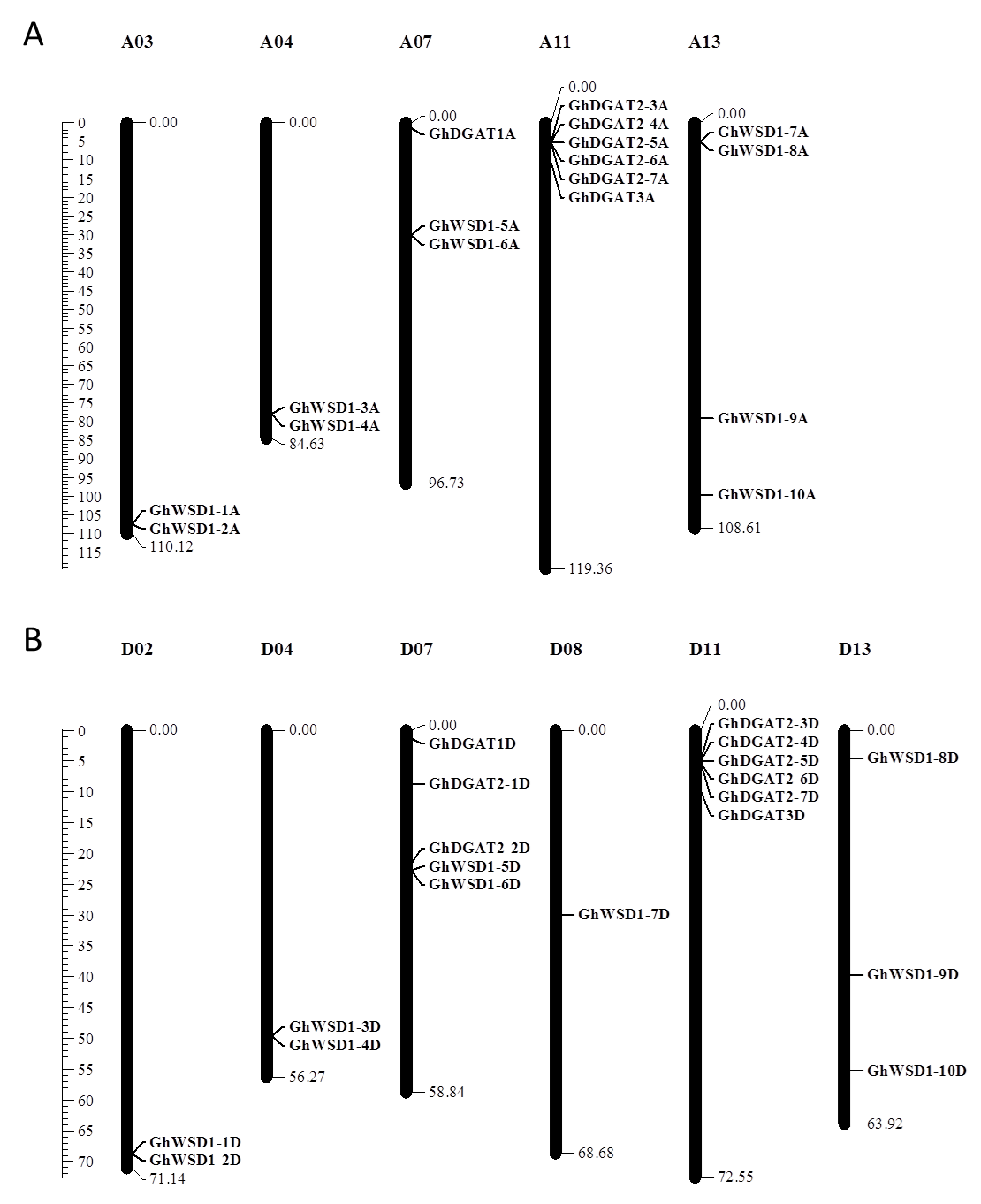

Supplement: Supplementary file 1 [file genes-12-01045-s001.zip › Fig. S1.tif]

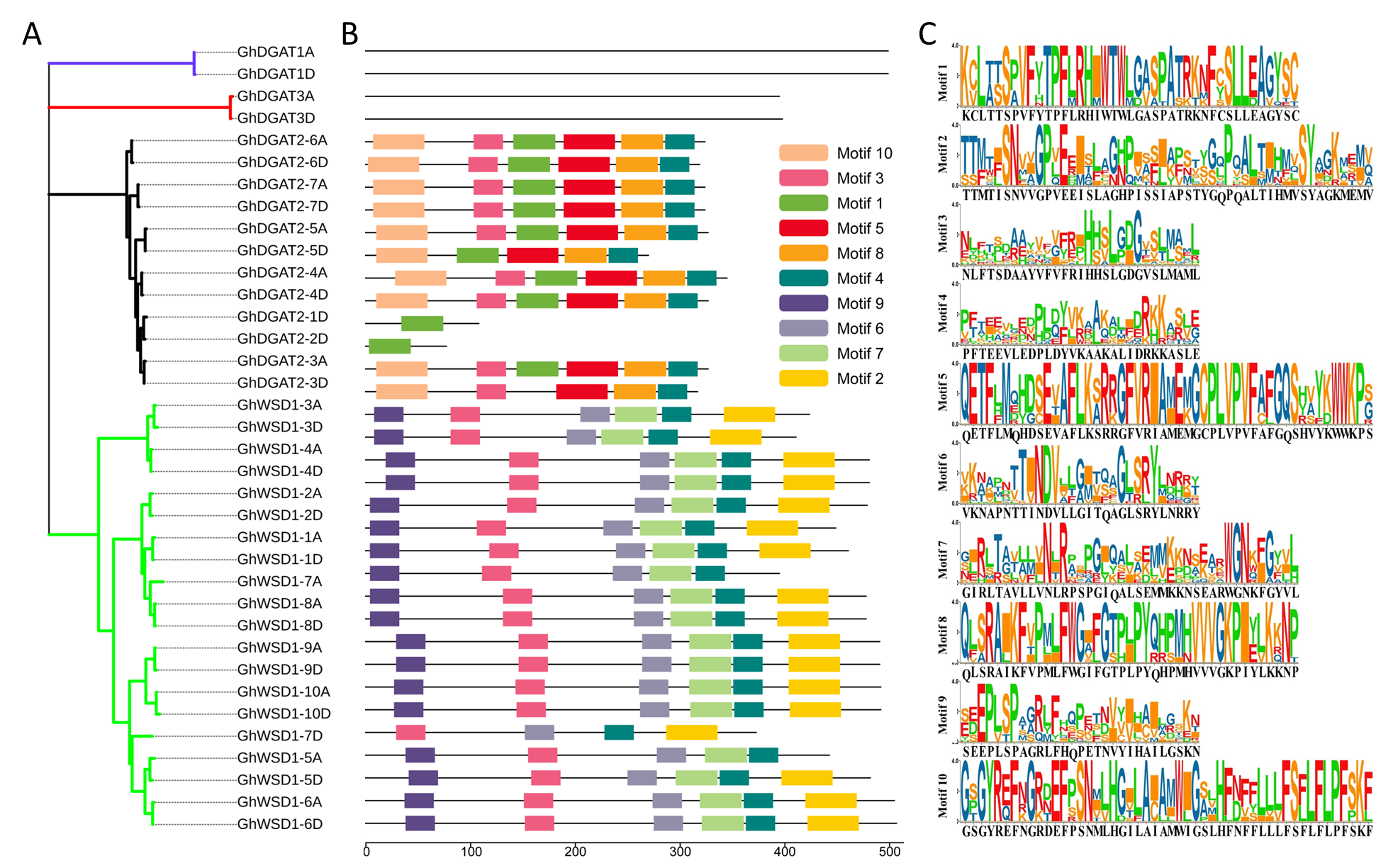

Supplement: Supplementary file 1 [file genes-12-01045-s001.zip › Fig. S2.tif]

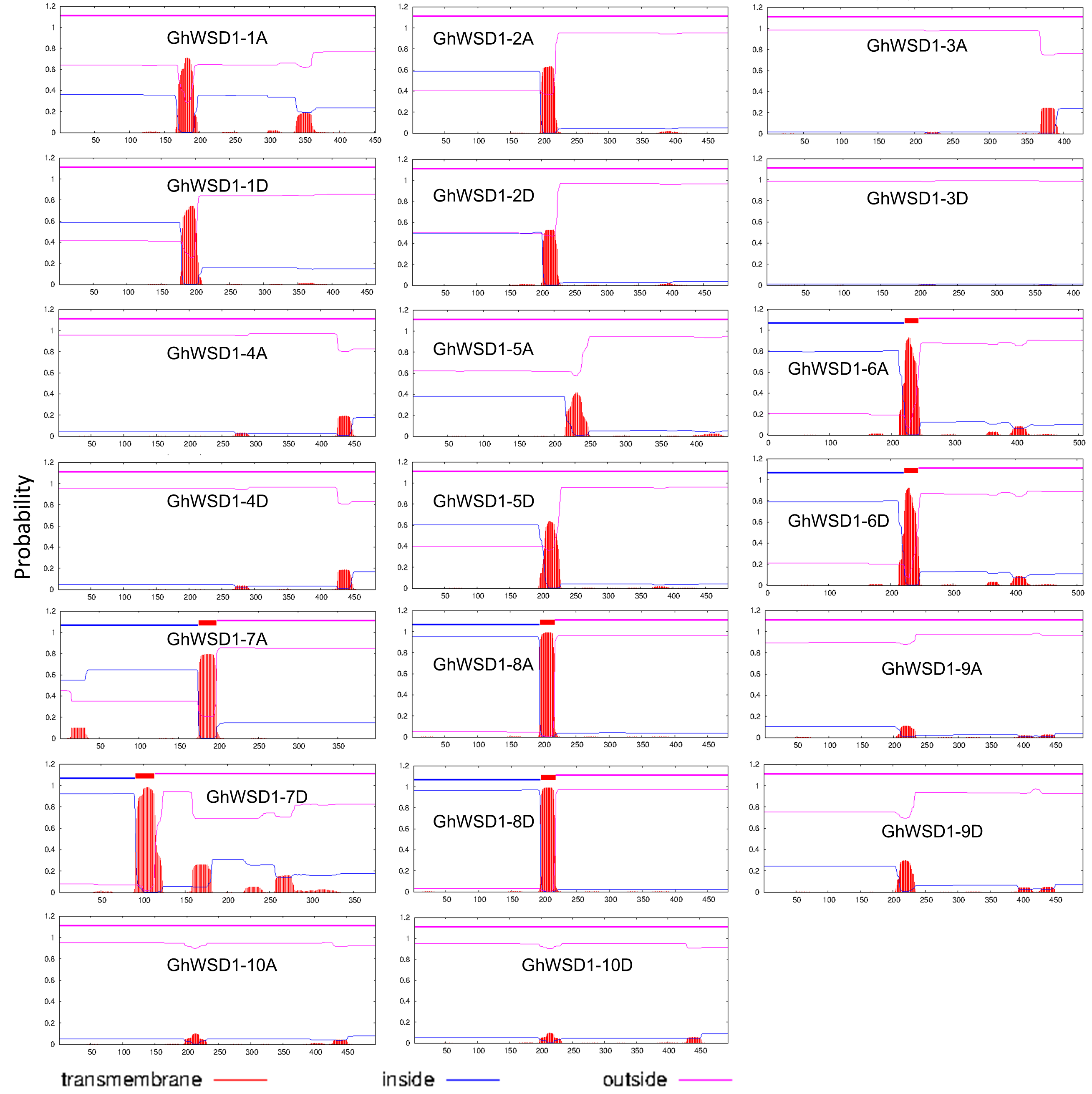

Supplement: Supplementary file 1 [file genes-12-01045-s001.zip › Fig. S3.tif]

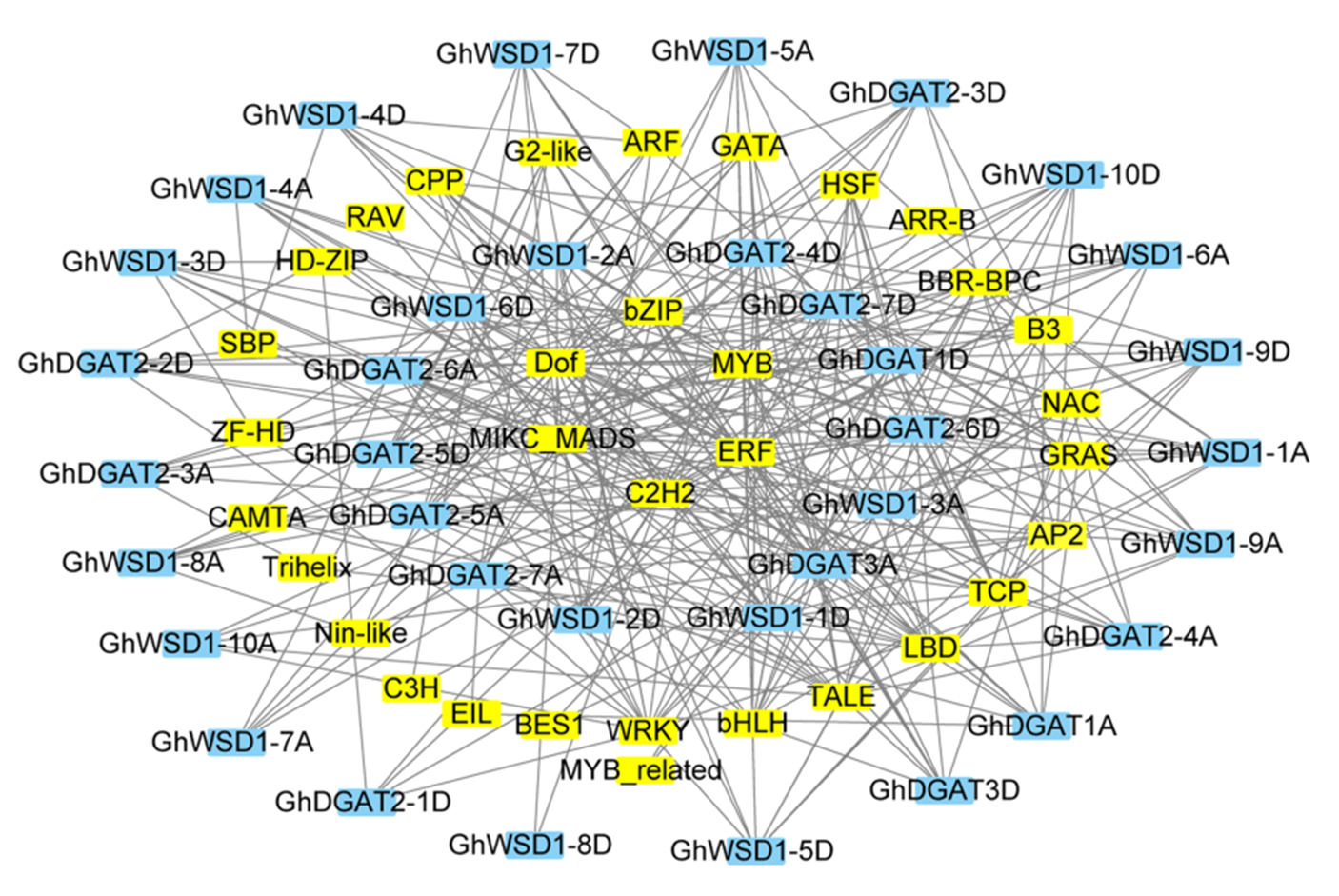

Supplement: Supplementary file 1 [file genes-12-01045-s001.zip › Fig. S4.tif]

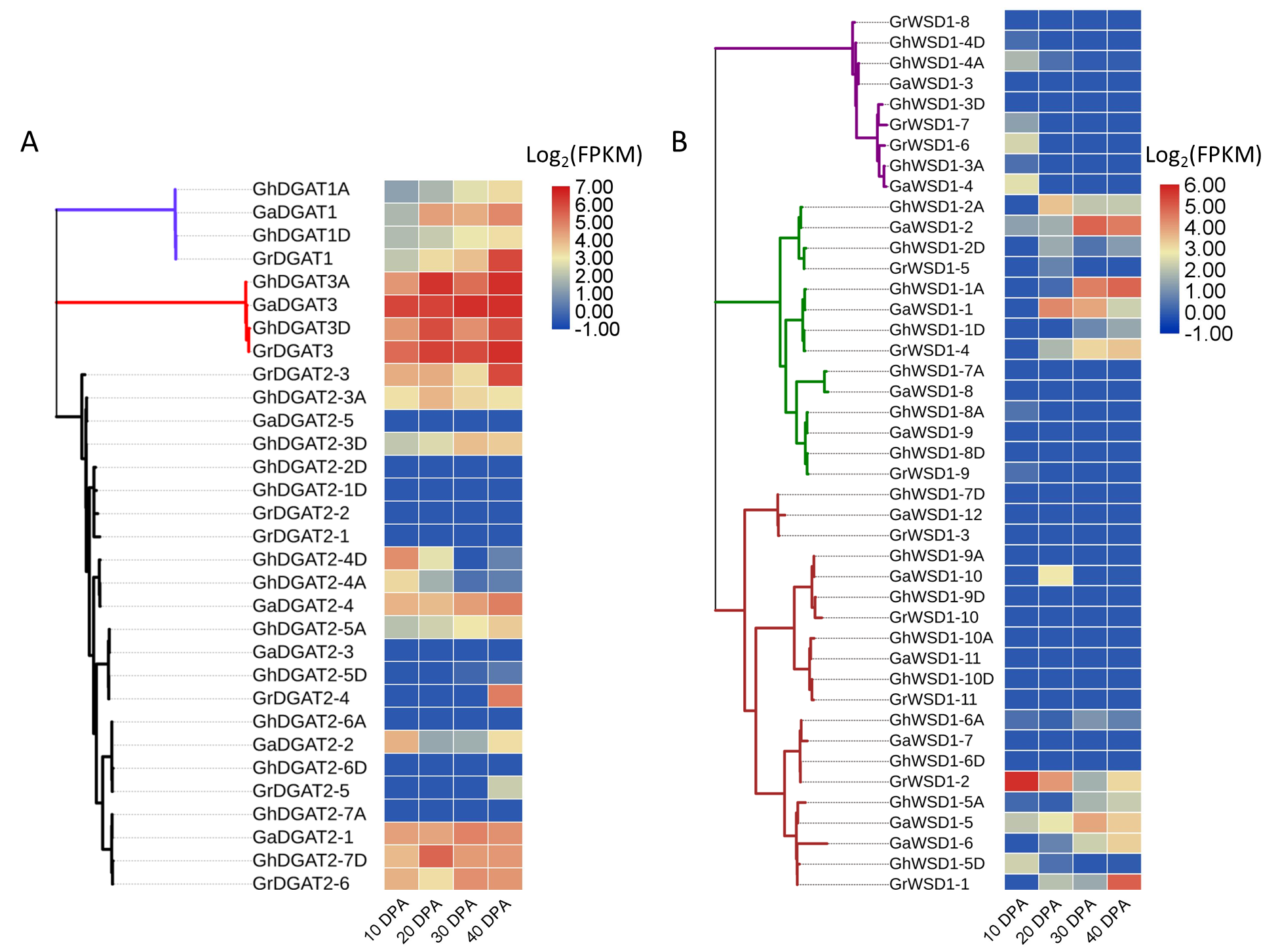

Supplement: Supplementary file 1 [file genes-12-01045-s001.zip › Fig. S5.tif]

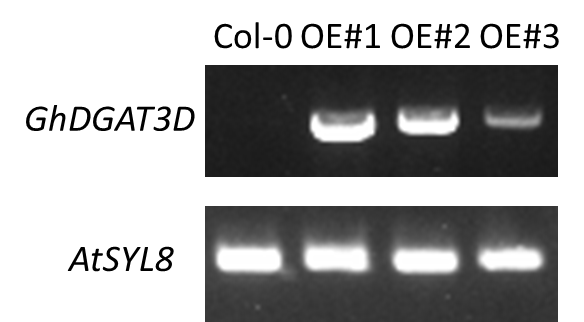

Supplement: Supplementary file 1 [file genes-12-01045-s001.zip › Fig. S6.tif]
